# Encapsulation of Protein-Polysaccharide HIP Complex in Polymeric Nanoparticles

**DOI:** 10.1155/2011/458128

**Published:** 2011-04-27

**Authors:** Ripal Gaudana, Varun Khurana, Ashwin Parenky, Ashim K. Mitra

**Affiliations:** Division of Pharmaceutical Sciences, School of Pharmacy, University of Missouri-Kansas City, 2464 Charlotte Street, Kansas City, MO 64108-2718, USA

## Abstract

The objective of the present study is to formulate and characterize a nanoparticulate-based formulation of a macromolecule in a hydrophobic ion pairing (HIP) complex form. So far, HIP complexation approach has been studied only for proteins with molecular weight of 10–20 kDa. Hence, we have selected bovine serum albumin (BSA) having higher molecular weight (66.3 kDa) as a model protein and dextran sulphate (DS) as a complexing polymer to generate HIP complex. We have prepared and optimized the HIP complex formation process of BSA with DS. Ionic interactions between basic amino acids of BSA with sulphate groups of DS were confirmed by FTIR analysis. Further, nanoparticles were prepared and characterized with respect to size and surface morphology. We observed significant entrapment of BSA in nanoparticles prepared with minimal amounts of PLGA polymer. Finally, results of circular dichroism and intrinsic fluorescence assay have clearly indicated that HIP complexation and method of nanoparticle preparation did not alter the secondary and tertiary structures of BSA.

## 1. Introduction

Protein-based therapeutics such as antibodies, blood derived products, and vaccines have been widely investigated in the past decade to treat a variety of disorders [1]. Development of a nanoparticulate-based dosage form of these molecules is still considered as a major challenge by scientists in the drug delivery field. Single emulsion (O/W), double emulsion (W/O/W), and emulsion polymerization have been widely employed to prepare nanoparticles. Except emulsion polymerization, the other two methods (single and double emulsion) employ organic solvents and sonication during nanoparticle preparation. Protein-based therapeutics tend to exhibit rapid denaturation and conformational change due to sonication and exposure to organic solvents [2, 3]. These molecules may aggregate and eventually lose their biological activity due to physical and chemical stress observed during formulation development, for example, exposure to organic solvents and sonication. These molecules may also denature or lose their biological activity during storage and lyophilization [4–6]. Sonication is employed to ensure homogeneous dispersion of an emulsion. However, sonication may result in large pressure and temperature gradient which may cause denaturation and aggregation of the protein molecule [7]. Moreover, sonication also causes generation of high shear force and free radicals which cause protein denaturation [7]. Organic solvents preferentially interact with nonpolar amino acids of protein via hydrophobic interactions. Normally, these nonpolar amino acids are present in the core of the protein structure. As a result, in presence of organic solvents, the native structure and conformation of the protein can be altered. This process may result in loss of biological activity of a protein molecule. Another crucial formulation-related limitation of protein molecules is their hydrophilicity. Due to their hydrophilic nature, these molecules often partition poorly into the polymeric matrix during encapsulation resulting in minimal loading in nanoparticles [1]. Due to poor loading of these molecules, a higher amount of polymer is needed to develop a formulation. Poly lactic-co-glycolic acid (PLGA) is one of the most widely employed biocompatible and biodegradable polymers utilized in the preparation of nanoparticles. However, higher amounts of PLGA can lower the stability of protein molecules as protein molecules were found to be unstable in presence of lactic acid and glycolic acid which are degradation products of PLGA [6, 8, 9]. 

Hydrophobic ion pairing (HIP) complexation based approach has gained wide acceptance in the delivery of peptide and protein based therapeutics [10–14]. In this approach, ionizable functional groups of a drug molecule are ionically complexed with a surfactant or polymer with oppositely charged functional groups. The resulting drug-polymer or drug-surfactant complex is known as HIP complex. Since the hydrophilic protein molecule exists in a complex form which is relatively hydrophobic, its partition into the polymeric matrix can be significantly enhanced during encapsulation [10, 15]. Protein and polymer (used for HIP complexation) primarily interact due to ionic interactions resulting in the formation of a HIP complex. The complex can dissociate in presence of oppositely charged ions. Further, HIP complexation would obviate the use of any covalent modification in proteins to impart these molecules more hydrophobicity. Covalent modifications may also result in irreversible loss in the biological activity of these molecules. Various studies have been performed in the past to understand the nature of protein-surfactant interactions. HIP complexation approach has been studied with various peptide and protein based therapeutics such as leuprolide, insulin, melittin, lysozyme, and so forth [10–13]. HIP complexation of protein-based therapeutics has been attempted to overcome various barriers associated with delivery of protein molecules such as bioavailability and stability [13, 16]. Moreover, HIP complexation can also impart conformational stability to the protein molecule [13]. 

HIP complexation of large protein molecules is challenging primarily due to following reasons. Large molecules usually contain many groups with opposite charges which may hinder the complexation process. So far, basic amino acids have been employed (mainly lysines and arginines) to form a HIP complex with anionic surfactant molecules. However, in large protein, aspartic acid and glutamic acid are also present on the surface in significant numbers which would repel the negatively charged complexing molecules. Second, in a large molecule, charge density plays a very crucial role. There is usually more surface area per charge in a large protein than for a small protein molecule. Hence, selection of a surfactant or polymer with an appropriate chain length is necessary to form the HIP complex. Activity of a protein molecule also depends on its secondary and tertiary structures. These structures are stabilized by various noncovalent interactions such as electrostatic interactions, hydrogen bonds, Van der walls forces, and hydrophobic interactions [17–19]. Hence, a complexing agent which would not perturb the secondary and tertiary structure of the protein must be selected. So far, various surfactant molecules have been selected to prepare HIP complex. In the present study, we have investigated HIP complex formation by employing dextran sulphate, a polysaccharide-based molecule. 

Bovine serum albumin (BSA) is a 66.3 kDa molecule. It is globular in shape and has been widely used as a model protein [20, 21]. Dextran sulphate, (DS, molecular weight: 9–20 kDa), a polysaccharide-based polymer, has been selected for complexation. In this paper, HIP complex of BSA with DS has been described. Solid in oil in water (S/O/W) emulsion method has been employed to prepare nanoparticles. After preparation, nanoparticles have been characterized with respect to particle size and surface morphology. Finally, the effect of HIP complexation and nanoparticle preparation on the secondary and tertiary structure of BSA has been studied by circular dichroism and intrinsic fluorescence assay, respectively.

## 2. Materials and Method

Materials: Bovine serum albumin, dextran sulfate sodium salt (molecular weight 9000–20000 da), Poly (DL-lactide-co-glycolide) (PLGA 85 : 15, molecular weight of 50,000–75,000 da), bicinchoninic acid (BCA), and copper sulphate were procured from Sigma Aldrich. Micro-BCA protein assay kit was purchased from Thermo scientific. All the solvents and other reagents of analytical grade were purchased from local suppliers and used as received without any further purification. Double distilled water (DDW) was used throughout the entire study.

### 2.1. Preparation of HIP Complex of BSA and DS

Stock solutions of BSA and DS were prepared in citrate buffer pH 4.4 and DDW, respectively. BSA consists of various basic amino acids (60 lysine and 26 arginine residues) while DS contains 2.3 sulphate groups per glucosyl residue. HIP complex was formed spontaneously as both the aqueous solutions were mixed.

### 2.2. Effect of Different Molar Ratios of DS to BSA on HIP Complex Formation

Stock solutions of BSA and DS were prepared as mentioned earlier. HIP complexes were prepared in different molar ratios of DS/BSA. The molar ratios studied were 0.29, 0.58, 0.87, and 1.15. These molar ratios represent the addition of different amounts of DS into previously prepared BSA solution (5 mg/mL in pH 4.4 citrate buffer). Once formed, HIP complex was vigorously vortexed for 3 minutes followed by centrifugation at 10000 RPM for 10 minutes to separate the supernatant. Uncomplexed BSA was measured in the supernatant using BCA assay. Percentage of complexed BSA was calculated according to the following equation:

(1) $$\begin{array}{cc} & \text{\%}\;\text{Complexed}\;\text{BSA}\\ & =\left[\frac{\text{Initial}\;\text{amount}\;\text{of}\;\text{BSA}-\;\text{amount}\;\text{of}\;\text{BSA}\;\text{in}\;\text{supernatant}}{\text{Initial}\;\text{amount}\;\text{of}\;\text{BSA}}\right]\\ & \;*100. \end{array}\;$$

### 2.3. Dissociation of BSA from HIP Complex

Dissociation of BSA from HIP complex was studied to characterize the nature of interaction between BSA and DS. Freeze dried complex containing 5 mg of BSA was accurately weighed and incubated in presence of DI water and aqueous solution containing 10 mM Na_2_HPO_4_. These solutions were vortexed and kept for equilibrium for 3 hrs at room temperature. After 3 hrs, these solutions were subjected to centrifugation and supernatant was collected. The concentration of dissociated protein in the supernatant was then measured with BCA assay.

### 2.4. FTIR Study

FTIR analysis of BSA, DS, and HIP complex was carried out with an infrared spectrophotometer (Perkin-Elmer, Waltman, MA). The samples were brought into intimate contact with the diamond crystal by applying a loading pressure. Samples were casted on diamond crystal top-plate of Attenuated Total Reflectance (ATR) accessory and scanned between 650–1800 cm^−1^. Spectra obtained using this device represents the average of 32 individual scan possessing a spectral resolution of 4 cm^−1^.

### 2.5. Preparation of Nanoparticles

PLGA 85 : 15 was used as a polymer to prepare nanoparticles. Nanoparticles were prepared by using solid in oil in water (S/O/W) emulsion method published earlier with minor modifications [15]. Briefly, 5 mg of BSA in complex form was used for preparation of nanoparticles. PLGA 85 : 15 was dissolved in methylene chloride. Two different ratios of BSA: PLGA 85 : 15 (1 : 5 and 1 : 10) were employed to prepare the nanoparticles. PLGA solution was gradually added to the earlier prepared HIP complex. Total volumes of methylene chloride and vortexing time were optimized to obtain S/O dispersion. About 4-5 mL of methylene chloride was required to completely disperse the HIP complex. Sonication was performed for about ≈3 minutes using tip sonicator (Fisher 100 Sonic dismembrator, Fisher Scientific) at power output of 25–30 W to obtain the fine S/O dispersion. To this S/O dispersion, external aqueous phase (30 mL, 1% PVA) was added followed by further sonication for ≈3-4 minutes. This procedure resulted in S/O/W nanoemulsion which was kept on a shaker bath at room temperature for 15–20 minutes followed by complete evaporation of methylene chloride using a Rotavap. Following evaporation, the nanodispersion was centrifuged for 50 minutes at 22,000 g. Nanoparticles were washed two times with DI water to remove any surface bound BSA and PVA. Similarly, blank nanoparticles were also prepared by employing only polymer (PLGA 85 : 15) in similar amounts.

### 2.6. Characterization of Nanoparticles

#### 2.6.1. Entrapment Efficiency of Nanoparticles

Entrapment efficiency was measured according to an earlier published protocol [20, 21] with minor modifications. Briefly, 1mL of nanosuspension was added to 9 mL of methylene chloride solution which was then vortexed for 10–15 minutes to dissolve the polymer completely. Later, this solution was subjected to centrifugation which resulted in formation of a protein pellet. Methylene chloride was carefully separated and the pellet was dissolved in 10 mL of PBS buffer. Concentration of BSA in the aqueous phase was measured using Micro-BCA assay. Absorbance from the samples were corrected by subtracting the absorbance from blank nanoparticles prepared using PLGA 85 : 15.

#### 2.6.2. Particle Size Measurement

Previously published protocol [15] was followed to measure the mean particle size and polydispersity of nanoparticles using a DLS instrument (Brookhaven Inst. Co., USA). Particle size analysis was carried out at an operating angle of 90°C and temperature of 25°C. A dilute sample of the nanosuspension was taken for particle size analysis, and at least three measurements of each batch were carried out.

### 2.7. SEM and TEM Analysis

For SEM analysis, freeze dried specimen was applied on a sticky carbon film positioned on an aluminum stub. Specimens were sputter coated with gold-palladium and observed with the field-emission SEM XL30 (FEI, Hillsboro, OR). For TEM study, a drop of nanosuspension was deposited on TEM cooper grid with carbon film. After drying, it was observed under Phillips TEM CM12 (FEI, Hillsboro, OR).

### 2.8. Evaluation of Secondary Structure of BSA after Dissociation from HIP Complex and Release from Nanoparticles with Circular Dichroism

HIP complex was dissociated in presence of 1 mL of 10 mM Na_2_HPO_4_ solution, and free BSA was quantified using BCA assay. Previously prepared PLGA nanoparticles were incubated in presence of 1 mL of 10 mM Na_2_HPO_4_ solution and kept overnight. BSA released from the nanoparticle formulation was quantified on the following day with BCA assay. Finally, standard solution of BSA was prepared in 10 mM Na_2_HPO_4_ solution and used as a control. Final concentration of each sample was adjusted to 0.05 mg/mL. Circular dichroism (CD) spectra were collected using Jasco 720 spectropolarimeter at room temperature. The spectra of all the samples were collected over a range of 200–250 nm with a cuvette of 1 cm path length at a scan speed of 20 nm/min. Data was further processed for blank subtraction and noise reduction and an average of three signals was recorded. All CD measurements are reported as ellipticities (*θ*, mdeg).

### 2.9. Evaluation of Tertiary Structure of BSA after Dissociation from HIP Complex and Release from Nanoparticles with Intrinsic Fluorescence Assay

Fluorescent measurements were carried out at room temperature with fluorescence spectrophotometer (Photon Technology International). The procedure to recover BSA after dissociation of HIP complex and from nanoparticles has been mentioned previously. Standard and test samples were prepared in 10 mM Na_2_HPO_4_ solution (final BSA concentration was adjusted to 0.1 mg/mL). We compared fluorescence spectra of standard with BSA obtained after dissociation from HIP complex and BSA released from nanoparticles. All samples were excited at a wavelength of *λ*_ex_ 295 nm, and emission spectra were collected between 310–400 nm. *λ*_ex_ 295 nm was chosen to selectively excite tryptophan amino acid of BSA. Quartz cells (12.5 L × 12.5 mm W) having 3 mL of sample capacity were used for measurement. Fluorescent emission spectra were recorded and are displayed in terms of relative fluorescence.

## 3. Result and Discussion

Proteins and peptides represent a rapidly growing class of therapeutic drugs with more than 200 biopharmaceuticals in the market and many more at different stages of development. Design of nanoparticle-based formulations for protein-based therapeutics has become a major challenge for drug delivery scientists because of poor encapsulation in polymeric matrix and rapid denaturation in presence of organic solvents and sonication [2, 3]. HIP complexation based approach can be explored to deliver peptide and protein-based therapeutics. It can overcome various stability related issues, enhance drug loading in nanocarriers and improve drug permeation across biological membrane [10–14, 22]. So far, HIP complex based approach has been only studied with small peptide and protein-based therapeutics. Hence, BSA was selected as a model protein in the present study because of its higher molecular weight (66.3 kDa) and well-known secondary and tertiary structure.

Isoelectric point (pI) of BSA is ≈4.5, and the protein consists of various basic amino acids (60 lysine and 26 arginine residues). Hence, we have slightly altered the pH of BSA solution and prepared stock solution of BSA at pH 4.4 in citrate buffer. Being hydrophilic in nature, these amino acids are mostly found on the protein surface. Amino groups of these basic amino acids are protonated based on the pH of surrounding medium. At this pH, HIP complex was formed immediately upon mixing of aqueous solutions of BSA and DS. This data confirms the importance of pH of the protein solution prior to HIP complexation. In general, it is crucial to understand the effect of pH on stability of protein molecule. One should also consider the possibility of other stability related issues which may arise by changing the pH of protein solution prior HIP complexation. 

**Figure 1:**
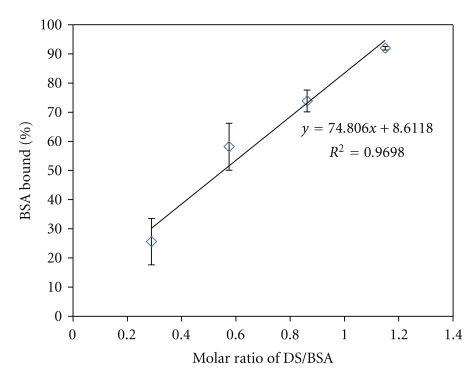
Effect of molar ratio of DS : BSA on HIP complex formation.

The effect of molar ratios of DS/BSA on HIP complex formation has been studied. We calculated the molar ratios based on the total number of lysine amino acids present on the surface of BSA (60 lysine amino acid). HIP complexes were prepared using the following molar ratios of DS/BSA (0.29, 0.58, 0.87, and 1.15). Theoretically, these molar ratios represent the amounts of DS added which was sufficient to complex with 15, 30, 45, and 60 basic amino acids of BSA.  Figure 1 shows the complexation of BSA with DS at different molar ratios.An excellent correlation is observed between increments in the molar ratio of DS/BSA with the amount of BSA complexed with DS (Figure 1).In fact at a molar ratio of 1.15, more than 90% of BSA molecules were ionically complexed with DS. This data clearly indicates the involvement of basic amino acids in the formation of HIP complex. 

**Figure 2:**
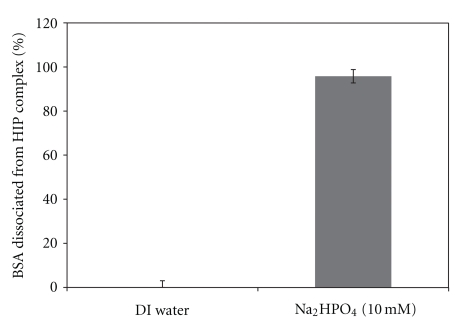
Comparative dissociation of BSA from HIP complex in the presence of DI water and 10 mM Na_2_HPO_4_ solution.

We also hypothesized ionic interactions as a driving force for complexation of BSA with DS. In order to confirm our hypothesis, we performed dissociation studies of the HIP complex in presence of oppositely charged ions (HPO_4_^−2^). Results of this experiment are shown in Figure 2. When HIP complex was incubated in DI water, no dissociation of BSA from HIP complex was observed. This could be due to low ionic strength of DI water. However, the presence of 1 mL of 10 mM Na_2_HPO_4_ solution caused complete dissociation of the HIP complex and the solution became clear. These data further confirm the presence of ionic interactions between amino group of basic amino acids in BSA and sulphate group of DS. Dissociation of HIP complex in presence of counter ions has also been reported by other investigators [13, 15].

**Figure 3:**
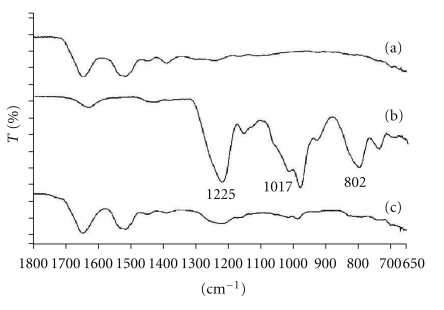
FTIR spectra of (a) BSA, (b), DS and (c) HIP complex.

FTIR study was performed to understand the nature of interactions between amino group of basic amino acids in BSA and sulphate group of DS. FTIR analysis was performed by other investigators to characterize ionic interactions between oppositely charged functional groups [12, 23, 24]. Due to overlapping shift in a FTIR spectrum, we did not follow peak shift associated with the protein. Instead, the interaction of sulphate group of DS was studied in the IR region. Following are the characteristic peaks of sulphate group of DS in the IR region: (a) 802 cm^−1^: S-O-S vibration, (b) 1017 cm^−1^: symmetric SOO^−^ stretching vibration, and (c) 1225 cm^−1^: asymmetric SOO^−^ stretching vibration. Appearance of these peaks in the IR spectra is close to previously published results [23–25]. Due to ionic interaction between amino and sulphate groups in HIP complex, the peak intensity of the sulphate group in the IR region may be attenuated significantly. Results of this study are shown in Figure 3. These results clearly indicate a significant reduction in the peak intensities of sulphate group in the IR region which again confirmed the presence of ionic interactions between amino and sulphate groups in the HIP complex. 

**Figure 4:**
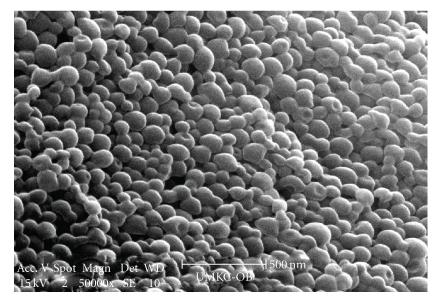
SEM images of nanoparticles.

**Figure 5:**
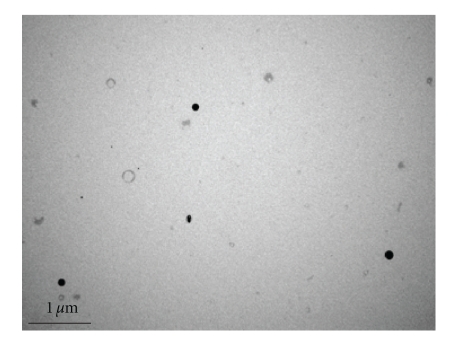
TEM images of nanoparticles.

We prepared nanoparticles of the complex using S/O/W emulsion method. This method of preparation offers significant advantages over conventional methods of nanoparticles preparation such as single and double emulsion method. In the conventional methods of preparation, protein is initially dissolved in an aqueous phase and later emulsified in the presence of an organic phase using sonication. Most protein denaturation occurs during this stage of nanoparticle preparation due to water-organic phase interface. Excessive stress during sonication process and generation of free radicals can cause protein unfolding and denaturation. In S/O/W emulsion method, protein-polysaccharide powder was employed in the preparation of nanoparticles instead of protein in solution form. Further, in the powder form, kinetic mobility of the protein is restricted compared to solution form [20, 21]. Moreover, complexation with DS would not only restrict conformational flexibility of BSA but would also impart additional *steric shielding* to the protein molecule. We optimized the total volume of organic solvent needed and the sonication time to prepare nanoparticles. Nanoparticles were also characterized with respect to particle size (Table 1) which range between 150–200 nm. SEM and TEM studies were performed to study the surface morphology. Results of these studies are shown in Figures 4 and 5, respectively. These results confirmed that particles have smooth surface and spherical shape. 

One of the important goal of the present study was to achieve higher encapsulation of BSA in nanoparticles by employing minimal amounts of polymer (PLGA 85 : 15). Nanoparticles were prepared by employing two different ratios of protein: PLGA (1 : 5 and 1 : 10). BSA entrapment in nanoparticles was more than 65% in both cases (Table 1). This data clearly shows a significant entrapment of BSA in PLGA matrix. As the amount of PLGA was increased to prepare nanoparticles, entrapment of BSA in nanoparticles was enhanced as well. This could be attributed to enhanced hydrophobic interactions of BSA in HIP complex with PLGA polymer. Due to these hydrophobic interactions, partition of BSA (in HIP complex form) in the polymeric matrix of PLGA was also significantly enhanced. 

**Figure 6:**
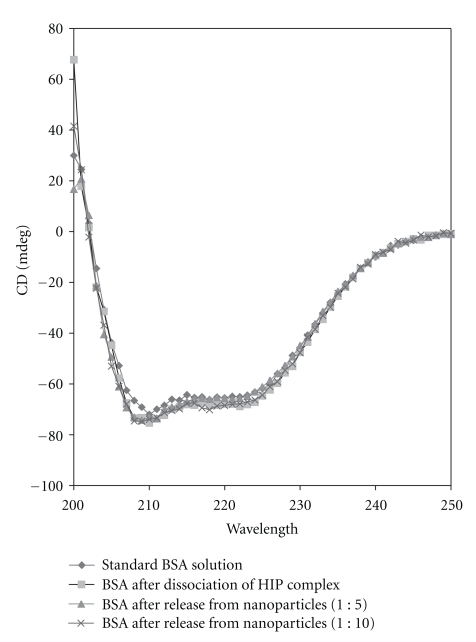
CD spectra of standard solution of BSA, BSA recovered after dissociation from HIP complex and BSA after release from different batches of nanoparticles.

The effect of HIP complexation and nanoparticle preparation on secondary structure of BSA was evaluated by CD spectra. Weak physical interactions such as electrostatic interactions, hydrogen bonds, Van-der-waals forces, and hydrophobic interactions stabilize secondary structure of the protein. During HIP complex formation, DS interacts extensively with BSA which involves abovementioned forces. So, it is quite possible that DS has altered the native conformation of BSA. Similarly, during nanoparticle preparation, powder form of BSA-DS complex was sonicated in presence of organic solvents. These processes could possibly denature BSA. CD analysis was performed to understand the impact of these formulation factors on secondary structure of BSA. Freshly prepared BSA in 10mM Na_2_HPO_4 _solution was selected as control.  Figure 6 depicts the CD spectra of standard BSA solution, BSA obtained from dissociation of HIP complex, and BSA released from both batches of nanoparticles. Results clearly show a significant overlap in peak shape throughout the region studied. This data also confirms that the secondary structure of BSA was not perturbed due to HIP complexation or treatment with organic solvent and sonication. Enhanced stability of BSA towards organic solvents and sonication may be explained by the following reasons. First, HIP complexation might have provided conformation stability and steric shielding to the BSA molecule. Moreover, with S/O/W emulsion method, the probability of protein denaturation has been significantly minimized compared to conventional method such as W/O/W emulsion method. In S/O/W emulsion method, protein molecules are encapsulated in the solid state relative to W/O/W emulsion method where solution form of protein is employed. In the solid state, the detrimental effect of sonication at water-organic phase interface is also minimal. 

**Figure 7:**
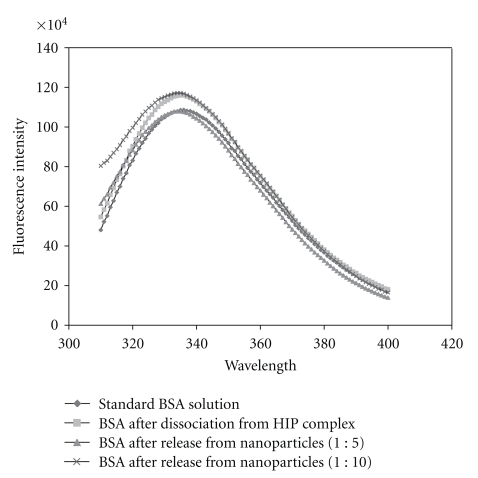
Intrinsic fluorescence assay of standard solution of BSA, BSA recovered after dissociation from HIP complex and BSA after release from different batches of nanoparticles.

We compared the intrinsic fluorescence spectra of freshly prepared BSA with BSA obtained after dissociation from HIP complex and BSA released from different batches of nanoparticles. BSA contains a buried tryptophan amino acid in its hydrophobic core. Fluorescence of tryptophan is extremely sensitive to polarity of its surrounding medium [26]. Changes in the fluorescence intensity, wavelength of maximum fluorescence emission, and quantum yield are accepted parameters to study tertiary structure of protein. Results of this study are shown in Figure 7. It is very clear from this data that intensity and wavelength of maximum fluorescence (335 nm) are similar in all the samples. This data confirmed that tertiary structure of BSA was not significantly altered following dissociation from HIP complex and also after release from nanoparticles. This result also corroborates with our previous CD spectra results where we have observed no significant change in secondary structure of BSA due to HIP complexation and nanoparticle preparation.

## 4. Conclusions

This study for the first time shows the feasibility of forming HIP complex of a large protein such as BSA with dextran sulphate as a complexing polymer. This study confirms the involvement of basic amino acids in the formation of HIP complexation. Dissociation studies of HIP complex in presence of oppositely charged ions (HPO_4_^−2^) as well as FTIR studies have revealed presence of ionic interactions between basic amino acids in BSA and sulphate groups of DS. We successfully prepared and characterized nanoparticles of BSA in HIP complex form using S/O/W emulsion method. SEM and TEM studies revealed smooth surface and spherical shape of nanoparticles. Significant entrapment of BSA in nanoparticles was obtained when low amounts of PLGA 85 : 15 was employed. Finally, CD analysis and intrinsic fluorescence data revealed that secondary and tertiary structures of BSA were not affected due to HIP complexation and nanoparticle preparation. HIP complexation approach can be employed to enhance loading of large proteins including antibody-based therapeutic molecules in colloidal dosage forms.

## Figures and Tables

**Table 1:** Particle size, polydispersity, and entrapment efficiency of different batches of nanoparticles. Values are given as means ± SD (*n* = 3).

Ratio of BSA to PLGA 85 : 15	Particle size (nm)	Polydispersity	% Entrapment efficiency
1 : 5	193.4 ± 3.1 nm	0.011	67.8 ± 8.6
1 : 10	201.6 ± 2.2 nm	0.010	79.7 ± 4.1
